# Sternal wound infections in cardiac surgery: effects of vancomycin prophylaxis

**DOI:** 10.1186/cc13551

**Published:** 2014-03-17

**Authors:** F Ampatzidou, M Sileli, C Koutsogiannidis, A Vlachou, A Baddour, G Drossos

**Affiliations:** 1 General Hospital 'G. Papanikolaou', Thessaloniki, Greece

## Introduction

Appropriate antibiotic prophylaxis plays a crucial role in preventing sternal wound infection after cardiac surgery [1]. In institutions with high prevalence of methicillin-resistant *Staphylococci*species, vancomycin prophylaxis is recommended either as a monotherapy or as an adjuvant agent [2]. In our study, we assessed sternal wound infection rates before and after the introduction of a vancomycin prophylaxis protocol.

## Methods

Twenty-six of a total 227 consecutive cardiac surgical patients, between July and December 2012, developed sternal wound infection (Group A). All of the patients received a standard empirical antibiotic prophylaxis. From January to July 2013, 308 patients underwent cardiac surgery (Group B). In this group, we applied a more restricted antibiotic protocol, considering the resistance patterns and the results of microbiological tests of group A. We also evaluated the results of MIC susceptibility testing of five antibiotics: oxacillin, linezolide, daptomycin, teicoplanin and vancomycin. In the new protocol the first vancomycin dose was given 1 hour before sternal incision followed by three additional doses (48 hours duration).

## Results

In group A, 26 patients (11.45%) developed sternal wound infection. Twenty out of 26 patients had staphylococcal infections characterized by high prevalence of oxacillin resistance while six patients had Gram-negative infections. In the vancomycin prophylaxis group (group B) we observed a significant reduction in the incidence of sternal wound infection rate (*P *< 0.01). Among 308 patients, six patients (1.94%) developed sternal wound infections, two of them caused by coagulase-negative oxacillin-resistant staphylococci while in four patients Gram-negative microorganisms were cultured. Mortality in infected patients was 0% in both groups.

## Conclusion

Appropriate antibiotic prophylaxis in cardiac surgery patients is of paramount importance in preventing sternal wound infections, resulting in dramatic reduction of postoperative morbidity and in significant economic benefits.
